# The Sexual and Reproductive Health Context of an Internally Displaced Persons' Camp in Northeastern Nigeria: Narratives of Girls and Young Women

**DOI:** 10.3389/frph.2021.779059

**Published:** 2022-01-13

**Authors:** Heather M. Marlow, Michael Kunnuji, Adenike Esiet, Funsho Bukoye, Chimaraoke Izugbara

**Affiliations:** ^1^International Center for Research on Women, Global Health Youth and Development, Washington, DC, United States; ^2^Department of Sociology, University of Lagos, Lagos, Nigeria; ^3^Action Health Incorporated, Lagos, Nigeria

**Keywords:** humanitarian, internally displaced persons (IDPs), reproductive health, Nigeria (northern), girls, young women

## Abstract

In humanitarian settings, ~35 million girls and young women of reproductive age (15–24) are in urgent need of sexual and reproductive health (SRH) information and services. Young women and girls in humanitarian contexts are particularly vulnerable to unwanted pregnancies, unsafe abortion, gender-based violence, and early and forced marriage. We sought to understand girls' and young women's experiences with unwanted pregnancy, abortion, contraception, sexually transmitted infections (STIs), gender-based violence (GBV), and forced marriage in an IDP camp in Northeastern Nigeria. We conducted 25 in-depth interviews with girls aged 15–19 (*N* = 13; 8 single and 5 married) and young women aged 20–24 (*N* = 12; 3 single and 9 married). All interviews were audiotaped, transcribed, translated, computer recorded and coded for analysis. The participants in our study fled from and witnessed violence to arrive in the IDP camp with little material support. Lack of necessities, especially food, has driven many to sex in exchange for goods or into forced marriages. This, in turn, leads to increased unwanted pregnancies and unsafe abortions. Participants had limited knowledge about contraception, and some information about SRH services available in the camp, but overall, knowledge and utilization of SRH services was low.

## Introduction

In 2021, 235 million people will need humanitarian protection and assistance accounting for 1 out of every 33 people worldwide (1). Eighty-two million people worldwide have undergone forced displacement due to political upheaval and conflict with more than half of them under the age of 18 (2, 3). The number of Internally Displaced Persons (IDPs) due to conflict and violence has dramatically increased in the last 10 years to 51 million new and existing IDPs and Africa is home to one-third of them (1, 4).

Humanitarian emergencies, including forced displacements and fragile contexts, heighten sexual and reproductive health (SRH) vulnerabilities, particularly for women and girls (5–7). For example, forced displacements and conflicts strain health systems and cause significant disruptions in access to critical SRH services. Conflicts constrain access to services and supplies; damage health facilities; elevate exposure to sexual violence and unsafe abortion; and increase risks for early and forced marriage, early childbearing, trafficking, and sexual exploitation, among other poor SRH outcomes (5–7). In humanitarian settings, ~35 million girls and young women of reproductive age (15–25) are in urgent need of SRH information and services (8). While the minimum health care package in humanitarian settings requires the inclusion of SRH services, use of and access to SRH services in many refugee contexts and situations of internal displacements in sub-Saharan Africa remains sub-optimal, especially for women and girls (6, 9, 10).

Over 2.1 million people in Northeastern Nigeria have been displaced by the Boko Haram insurgency (2, 3). According to UNHCR, 2.3 million girls and 1.6 million women need humanitarian assistance in Northeastern Nigeria (11). Many displaced persons fled violence and settled in IDP camps in the region. Borno State is at the epicenter of the displacement crisis and hosts a total of 1.5 million IDP (12). In Borno State, 54% of IDPs live in camps or camplike settings and 91% have been displaced two or more times (12). Women and children in these IDP contexts are disproportionately impacted and particularly vulnerable to sociocultural, economic, and SRH challenges. The most recent Demographic and Health Survey (DHS) data from Borno state indicates low use of contraceptives at 6.2% among married and sexually active women of reproductive age (15–49) and an unmet need of 12.2% for 15–19 year olds and 16.1% for 20–24 year olds (13). Gender based violence is also prevalent in the state at 35% of 15–49 year olds experiencing physical violence and 6.9% sexual violence; however, this is likely higher among IDPs and underreported in the camp (11, 13). Maternal mortality is extremely high in Northern Nigeria at 709 maternal deaths per 100,000 live births compared to <20 in developed nations (14). One of the leading causes of maternal mortality in Nigeria is unsafe abortion which has been on the rise in Northeastern Nigeria due to the Boko Haram insurgency (14, 15). A 2018 UNOCHA report indicates that ~1.7 million women and girls in Borno State require life- saving and essential health services (16). An estimated 600,000 of these displaced women and girls are of reproductive age and have major needs for SRH services, contraception, emergency obstetric care, and treatment for sexual and gender-based violence (16).

As part of a program to improve SRH services for young women and girls in humanitarian contexts, we sought to understand girls' and young women's experiences with unwanted pregnancy, abortion, contraception, STIs, gender-based violence (GBV), and forced marriage in an IDP camp in Northern Nigeria. This IDP camp consists of 10,000 households and a population slightly more than 50,000, of which one fifth are children and adolescents. The available SRH services in the camp include two health facilities (UNFPA and UNICEF), GBV services provided by Grassroots Initiative for Strengthening Community Resilience (GISCOR), UNFPA, and Danish Refugee Council (DRC), and two safe spaces (UNFPA and UNHCR). Other providers in the camp include Nigeria National Emergency Management Agency (NEMA; food distribution), Borno State Emergency Management Agency (SEMA: camp administration), IOM (camp coordination and management), GISCOR (protection and referral services), DRC (protection and livelihood), Center for Integrated Development and Research (CIDAR) (water and sanitation), UNFPA (protection, health, and skills acquisition), and UNICEF (health). There are 12 water points in the camp and 285 latrines. Tribes represented in the camp include Hausa, Gamargu, Kanuri, Fulani Shuwa and Wula-Wula.

## Materials and Methods

We conducted 25 in-depth interviews with single and married girls and young women ages 15–24. Criteria for inclusion were written assent or consent and parental/guardian consent where needed, age, marital status (single or married), and current resident of the IDP camp. Girls and young women were recruited by a local non-governmental organization working on SRH issues in the camp. Girls and young women involved in programs run by the NGO and known to workers co-located in the camp were asked to participate. The recruiters visited girls in their tents/temporary shelters. These workers/recruiters were trained on study procedures and how to approach girls and young women and their guardians or parents about the study. Girls and young women were asked if they were willing to participate in the interview. After providing assent or consent and obtaining parental/guardian consent where necessary, interviews were arranged in a private location with visual and audio privacy. Interviews were conducted by a trained interviewer. The interviews were conducted in local languages or in English, depending on the participants' preference, and were recorded. The participants did not receive renumeration, but hygiene kits containing soap and sanitary pads were given to participants at the end of interviews as incentives.

A semi-structured in-depth interview guide was developed in English and translated into local languages. The in-depth interview guide covered the following topics: conditions in the camp, understanding of SRH and available services in the camp, personal experiences related to unwanted pregnancy, sexuality education, contraception, abortion and post-abortion care, STIs, gender-based violence, forced/child marriage, maternal care services, and opinions about how to improve available services. Interviews lasted 45 mins to an hour, were recorded, transcribed, and translated into English where necessary. The study methods were approved by The National Health Research Ethics Committee of Nigeria and the International Center for Research on Women institutional review boards.

All transcripts were read in English for initial code identification. After reading the transcripts, a codebook was created with codes and their definitions. It included deductive codes from the in-depth interview guide as well as inductive codes emergent from the data. All in-depth interview transcripts were uploaded into NVivo 11 and coded by the first and second authors using the codebook (17). Once all transcripts had been coded, the first author reviewed all coding reports in NVivo and grouped codes into themes. Coded data were then analyzed by the demographic recruitment criteria of age and marital status to understand similarities and differences. The unit of analysis was the individual.

## Results

We interviewed 13 girls aged 15–19 and (8 single and 5 married) and 12 young women aged 20–24 (3 single and 9 married). One respondent had attended some primary school and 11 had attended some qu'ranic (Islamiya) school in the camp. The respondents lived in the camp from 2 to 7 years. None of the respondents received any sexual health education. Respondents learned about sexual and reproductive health from the UNFPA or Alima clinics (NGO clinic in Maiduguri), the hospital in Maiduguri, or from other women in the camp.

### Arrival at the Camp

Interview data highlighted the violent and traumatic events that preceded the respondents' relocation to the IDP camp. In their accounts, respondents recalled the peaceful and productive lives they lived before they arrived the camp. Many of the respondents were farmers, students, or small business owners. Participants' recollections of their pre-displacement lives centered on the peaceful existence they led, their access to basic essentials, including food and health services, and the strong social support systems they enjoyed. However, following attacks on and pervasive insecurity in their communities, the respondents were forced to flee, causing disruptions in all aspects of their lives. Narrative data indicated the loss and trauma experienced by the respondents following these attacks. One young woman described this:

*We were displaced from our homes by Boko Haram, they killed our parents, husbands, children, friends, wives. It was in my presence Boko Haram killed my brother and they went with his wife and my stepmom*. (IDI #1, Single, Age 18)

Security is an ongoing concern of respondents, due to continued activity of Boko Haram in the area. Previously, there were soldiers in the camp, but they have since been replaced by the Civilian Joint Task Force (JTF). Some respondents said they have security, but many believed it is not enough, and they still heard gunshots and feared Boko Haram attacks.

### Camp Conditions

Upon arrival in the camp, respondents and their families found little support for housing, food, and ways to make a living. The camp living conditions were difficult, as most people lived in tents or makeshift shelters, they did not have access to a water source nearby, latrines were far from where they lived, and food was scarce. Respondents' main concerns were lack of food, clothing, security, lack of money/job, shelter, education, and water. These conditions left many IDPs dependent on food rations, which were not sufficient. One woman explained:

*[The] government is trying [with] food distribution, although the food is not enough. [For] some people, it takes only 2 weeks, and the food is finished. [For] some people the food does not last up to a week and the food is finished. NGOs also used to support us with food. It is called Hajiya Bawa Kolo or SEMA. They are the only ones giving us food in this camp*.

(IDI #15, Married, 18 years old)

Many respondents reported that they were unable to buy additional food because they did not have a way to make a living. Men left the camp to collect firewood to sell but were vulnerable to violence from Boko Haram outside of the camp. Women knit caps for 2,000–3,000 Naira ($4–7 USD) to sell and purchase food. Yet, none of these strategies covered families' basic food needs. One respondent described this:

*My husband goes to look for money because we do not have a farm to cultivate. But we are really suffering because we hardly feed well. Our main problem is money. We do not have food as an adult, [but] even if we are hungry, we cope. But the children cannot cope*.

(IDI #17, Married, Age 20)

### Poor Sexual and Reproductive Health Outcomes and Lack of Resources

Respondents attributed poor sexual and reproductive health outcomes such as unwanted pregnancy, STIs and gender-based violence to underlying issues, such as lack of food and money, parents encouraging sex in exchange for food/money, and forced marriage, precipitated by the vulnerable situations IDPs found themselves in due to their displacement to the camp. Several respondents cited the lack of money or food as drivers of young people exchanging sex with older men. Two respondents described this:

*The cases of unwanted pregnancy are very high in the camp because the young people follow men and sleep around with them because they need money to feed themselves*.

(IDI #21, Married, Age 24)

*In this camp, STIs and HIV can easily [spread] when people sleep around because of hunger*.

(IDI #5, Single, Age 15)

Another young woman described that parents encouraged their daughters to exchange sex for money thus leading to unwanted pregnancy:

*Unwanted pregnancy is common in this camp. If the parents are not good, they will advise you to follow men so that you will bring money for them. In the process you will be pregnant*.

(IDI #14, Married, Age 15–19)

Unmarried girls with unwanted pregnancies were then too ashamed to go to the camp clinic:

*Those that [have] unwanted pregnancies go to clinics in town. They [do] not [feel] free to go to the camp clinic because of shame. [They do not want] people [to] hear that they are pregnant. Poverty has made so many ladies turn to sex for survival. [This] has brought about unwanted pregnancy and abortion*. (IDI #7, Single, Age 20)

Forced early marriage was cited as another consequence of lack of resources and a catalyst for gender-based violence. The main causes of forced early marriage as cited by respondents were the difficulties the parents face to care for their children due to lack of food and money. Therefore, they married their daughters at a young age, but as several respondents mentioned, this can also lead to gender-based violence. Several young women attributed gender-based violence to their own or others' early, forced marriages. They described this:

*The causes of GBV in this camp are parents, when they force their daughters to marry early and at a young age. As a young lady, GBV is not a good thing, and a girl should be mature enough before getting married*. (IDI #5, Single, Age 15)

*I am married but I had a problem with my husband and that's why I came back home. He used to beat me. I couldn't endure the beatings, so I came to [my home]. I have one daughter. I was forced to marry my husband. I don't know any GBV service available in this camp. I have never used any gender-based violence service in this camp*.

(IDI #15, Married, Age 18)

*There is child marriage. Children are forced into marriage because of money. Parents force their children into marriage*. (IDI #11, Married, Age 15–19)

Other precipitating causes of gender-based violence described by respondents were drug use, and disagreements about food or money.

### Challenges Faced by Younger, Unmarried Women

Younger women in our study were not only vulnerable to unwanted pregnancy and forced marriage, but also felt contraceptive services were only for married women, and instead sought abortions. Respondents mentioned that for younger women who are not married, it is harder for them to access contraception. One participant described this:

*[A] barrier to girls accessing contraception [is that] they are not married*.

(IDI #15, Married, Age 18)

Others said that young, unmarried girls have abortions nearby the camp because the camp clinic does not provide abortion services. Respondents reported that young girls have abortions in secret either outside of the camp or self-induced. Those seeking to abort keep it to themselves, unless there is a complication, in which case they can receive post-abortion care at a camp clinic. Several respondents described this:

*Girls who do abortion do it outside the camp because the camp clinic will not do any abortion for them. They do it without the consent of their parents. All they need is advice and a source of income*. (IDI #6, Single, Age 17)

*There are cases of abortion here in the camp. Young girls do abortion, but they hide doing it. No one knows, [unless] they fall sick. There is a place outside the camp*.

(IDI #11, Married, Age 15–19)

None of the respondents mentioned the abortion methods used at the abortion services outside of the camp nor whether the services are provided in a medical or non-medical setting, nor the type of practitioner. Some respondents also said that young women took abortion pills, metronidazole, Flagyl, and salt to induce an abortion. One respondent mentioned complications and death related to (presumably unsafe) abortion:

*Young women that abort their pregnancies do get problems and complications, which may sometimes lead to death*. (IDI #5, Single, Age 15)

### Married Women and Contraceptives and Childbirth

Of the ten respondents who knew about contraceptive methods or had used them, most were married women. Only one young woman interviewed knew about condoms and many girls and young women had no knowledge of contraceptives or condoms. Married women discussed contraceptive methods as useful for child-spacing, but that you should agree with your husband before initiating a contraceptive method. These women explained:

*My beliefs about contraceptives are good. It gives you child spacing. Those who should use them are those that need spacing*. (IDI #15, Married, Age 18)

*It is good to go and get family planning injection if you agree and if your husband agrees too, you can go and take [the] injection or they will insert rubber inside you*.

(IDI #8, Single, Age 18)

Several married women mentioned that the UNFPA clinic provides contraception.

Most women who knew about STIs were also married women, but overall, knowledge was low. Eight women knew of STIs and all of them knew of HIV and one woman also knew of gonorrhea from the radio. Of the women who knew about HIV, they correctly knew that it was transmitted sexually from unprotected sex. One respondent explained:

*Sexually transmitted infections are caused when a man and woman meet without any form of protection. Types of STIs are HIV, gonorrhea. If a man has it, he can infect a woman, and a woman too can infect a man. I have not heard of any information about STIs in this camp, but I heard it from a radio station. I don't know of tests for STIs available in the camp and also I have not heard of any treatment for STIs in this camp*. (IDI #15, Married, 18 years old)

Six respondents also mentioned “toilet infection” but were confused whether it was an STI or not. They said they were counseled by NGO and hospital workers to clean the toilet before using it, so that they would not get an infection. One respondent explained:

*There is something called infection in toilets, it is a dangerous thing. But there are drugs and treatment in the camp clinic for free. NGO workers went around telling us to be clean and use water to wash our toilets before using them. Before I use the toilet, I wash it*.

(IDI #20, Married, 20 years old)

Married women who gave birth in the camp had positive experiences with the Alima and UNICEF clinics, even when their cases were complicated. The women mentioned that they get adequate care that is free and that if there was a problem, they were quickly referred to the hospital. Two married women described this:

*The maternal services here are very good. They take care of us from when we get pregnant till when we give birth, and their services are free*. (IDI #17, Married, Age 20)

*Maternal services in the clinic are free and the camp clinic offers free medical attention for women and our children. The camp clinic gives us all the attention we need, and the services are free from pregnancy to delivery. The UNICEF clinic has helped us a lot*.

(IDI #18, Married, Age 19)

Other women delivered elsewhere due to the camp clinics being closed or because there were no clinic personnel available when the woman went into labor. Some women delivered at home and never had the intention of delivering in a health facility. These women explained:

*When a woman is in labor and goes to the clinic, you don't find the health personnel, because they don't come to work on time. It happened to me. When I [was ready to give] birth, we went to the clinic around 9 am but they were not around. So, we had to go out to Alima clinic where I gave birth*. (IDI #15, Married, Age 18)

*All my pregnancies were easy for me. I gave birth to all of my children at home. All of my labor was at night and the health workers here close by 5 pm*. (IDI #12, Married, Age 24)

## Discussion

Most participants fled the Boko Haram insurgency to arrive in the camp and witnessed or experienced tremendous violence along their journey. Although trauma and violence caused by the insurgency were not the focus of this research, it remains an underlying issue affecting all IDPs and must remain an integral part of services, especially mental health services, as part of an integrated package of healthcare in the camp. Mental health problems are associated with sexual risk-taking and inconsistent contraceptive use, leading to unwanted pregnancies, STIs, and unsafe abortion (18). These traumas are perpetuated by continued insecurity in the camp and frequent attacks on IDPs venturing outside the camp to earn a living. The greatest concern of IDPs interviewed was lack of food and many are forced to leave the camp to farm or earn small amounts of money to purchase food, further exposing them to Boko Haram violence. In a recent OCHA survey of IDP needs, food and undernutrition were of the greatest concern and were the greatest needs (19). The Covid-19 pandemic has exacerbated undernutrition and access to food for IDPs in Nigeria, along with increased poverty.

Lack of food and poverty affect girls and young women's sexual and reproductive health in that it can lead to exchange of sex for food or money. Interviewees discussed how lack of food or money to buy food drove them to exchange sex for food or money and, that parents send their daughters to do the same, or into forced/early marriages. Internally displaced girls and young women in other settings face similar vulnerabilities of prostitution, child trafficking, and sexual abuse (20, 21). These sexual encounters leave girls and young women extremely vulnerable to unwanted pregnancies, unsafe abortion, STIs, and gender-based violence.

The young women and girls interviewed discussed how sex in exchange for food or money and early/forced marriage led to a myriad of negative SRH outcomes. Several participants talked about how “following men” for food leads to unwanted pregnancy, which in turn leads to unsafe abortion. The pregnancies among younger unmarried women and girls brought shame due to their age and marital status, thus they were reluctant to go to camp clinics for pregnancy services. The underutilization of health services by girls and young women due to shame, embarrassment, fear of retribution, and social rejection has been documented in other refugee and IDP camps in Africa (22). There were no induced abortion services available in the camp (only post-abortion care), so young women instead turned to providers outside the camp or to unproved or dangerous methods, such as salt or Flagyl, and to keep their abortion secret. These types of unsafe abortions and abortion methods are common among young, vulnerable girls and women, especially where abortion is illegal (as is the case in Nigeria) or restricted (23, 24). Lack of food and resources also leads families to marry their daughters early, thus exposing them to early pregnancies, STIs and gender-based violence.

Several women reported experiencing gender-based violence or witnessing violence in their or other's households in the camp. The root causes of GBV are complex and multiple, but one woman experienced forced marriage and subsequently GBV as a result, and other women said that violence was perpetrated due to partner drug use and disagreements about food or money. Aham-Chiabuotu et al. (21) in their study of married couples in an IDP camp in Northeastern Nigeria found that “men masked their failures and inability to cope with the challenges of displacement by being physically violent, especially toward their wives, excessive alcohol intake, womanizing, or even marrying more wives”. Violence and drug and alcohol consumption are common in IDP settings (25–27) with negative consequences for vulnerable girls and young women.

Although most participants had very little formal education and no sexuality education, a number of women did have information about contraception and STIs. Married women knew of several contraceptive methods and reported using them, especially for child spacing, as well as some knowledge about STIs. They learned about STIs and obtained contraceptive methods from the UNFPA clinic in the camp. Unmarried women were less likely to have knowledge about STIs or contraceptives or to access contraceptive services because of the taboo of sexual activity outside of marriage, even though unmarried girls and young women in the camp were likely sexually active and some may be exchanging sex for food or money.

The married women who had given birth reported positive perinatal and birth experiences at the Alima and UNICEF clinics. Participants liked that the services for women and children were free and convenient, and even in the case of a serious complication, the woman was referred to the hospital and had a positive outcome. Some women preferred to give birth at home and several women complained that the maternity services were closed at the time they were ready to give birth and that they had to give birth elsewhere.

Our findings underscore that the drivers of poor SRH are related to underlying issues such as food scarcity, insecurity, and lack of employment. UN, government, and other non-governmental organizations are working to address issues of social protection, food, and livelihoods, that until addressed, will continue to influence families' options for girls and young women with negative outcomes for their SRH (3, 11, 19). Basic sexuality education for girls and young women could improve SRH knowledge and information; and when combined with skills building and empowerment, have been shown successful at improving SRH for young people in refugee settings (28). Underutilization of services such a contraception, STIs, safe abortion care, and GBV are in part due to perceptions that they aren't relevant (e.g. contraception for unmarried girls/young women), stigma related to unmarried girls or young women being sexually active, stigma of unwanted pregnancies among young, unmarried girls and young women, social stigma of seeking an abortion or post-abortion care, and lack of awareness of available STI and GBV services. Underutilization of available SRH services in humanitarian settings has been widely documented (7). More needs to be done to address these social perceptions and drivers of underutilization in this camp. The most mentioned and praised services in the camp are perinatal and maternal care for pregnant women and their children. These services should be studied more in-depth to understand what makes them successful in this setting and to apply their successes to those underutilized services.

Our study has several limitations. Information was missing on the exact ages of some participants, but we did record that they met the inclusion criteria for the age range. We did not collect information on demographics beyond those needed for the inclusion criteria. Information about pregnancies and parity would enrich the understanding of women's motivations behind use of available camp SRH services. The findings from this study should not be generalized beyond the camp. However, the findings are useful to understand girls and young women's SRH experiences in the camp.

This study highlights the precarious situations in which young IDP girls and women live, particularly related to food and resources. Due to the ongoing violence, underlying trauma and poverty, girls and young women in the camp are forced into sexual relationships and early marriages before they are ready. These circumstances in turn lead to poor SRH outcomes for girls and young women, including unwanted pregnancies, STIs, gender-based violence, and unsafe abortion. Although there are services available to them in the camp, many young women and girls are reluctant to access them due to shame and stigma. More integrated services must address food insecurity and lack of resources as drivers of early sex and forced marriage which in turn lead to poor SRH outcomes in this setting.

## Data Availability Statement

The datasets presented (qualitative interview transcripts) in this article are not readily available due to participant privacy. Requests to access the datasets should be directed to Heather Marlow, hmarlow@icrw.org.

## Ethics Statement

The studies involving human participants were reviewed and approved by the National Health Research Ethics Committee of Nigeria and the International Center for Research on Women IRBs. Written informed consent to participate in this study was provided by the participants' legal guardian/next of kin.

## Author Contributions

MK, AE, and FB supervised all data collection and data quality. MK and HM coded transcripts and all authors reviewed coding and themes for analysis. HM led writing with ongoing inputs from all authors. All authors reviewed manuscript drafts, conceptualized the study, and contributed to study protocols and procedures.

## Funding

This work was carried out with the aid of a grant from the International Development Research Centre, Ottawa, Canada.

## Conflict of Interest

The authors declare that the research was conducted in the absence of any commercial or financial relationships that could be construed as a potential conflict of interest.

## Publisher's Note

All claims expressed in this article are solely those of the authors and do not necessarily represent those of their affiliated organizations, or those of the publisher, the editors and the reviewers. Any product that may be evaluated in this article, or claim that may be made by its manufacturer, is not guaranteed or endorsed by the publisher.
